# The clinical impact of antimicrobial resistance genomics in competition with she-camels recurrent mastitis metabolomics due to heterogeneous *Bacillus* *licheniformis* field isolates

**DOI:** 10.14202/vetworld.2017.1353-1360

**Published:** 2017-11-20

**Authors:** Nesreen Allam Tantawy Allam, Doaa Sedky, Enshrah Khalil Mira

**Affiliations:** 1 Department of Parasitology and Animal Diseases, Veterinary Research Division, National Research Centre, Dokki, Giza, Egypt; 2 Department of Food Hygiene, Animal Health Research Institute, Agriculture Research Centre, Dokki, Giza, Egypt

**Keywords:** 16S rRNA gene, antimicrobial resistance, *Bacillus* species, Camelidae, Egypt, probiotics, recurrent mastitis, *rpo*B gene

## Abstract

**Background and Aim::**

Recently, cases of mastitis refractory to treatment have been reported frequently. There are limited routine laboratory investigations on Camelidae infections. Mastitis has been estimated to affect more than 25% of lactating she-camel with up to 70% milk loss. The details of *Bacillus* spp. pathogenesis in mastitis are not yet fully described. The present study is the first detailed phenotypic and genotypic characterization of *Bacillus licheniformis* isolates from recurrent mastitic she-camels with sepsis in Egypt.

**Materials and Methods:**

The udders of 100 she-camels were investigated, samples collected from smallholders’ farmers in 10 localities within three governorates in Egypt: Marsa Matrouh, Giza, and Sharkia governorates. The pathogens ascend from udder inducing abortion at different trimesters of pregnancy. Polymerase chain reactions-mediated proofs of identity were applied for diagnostic and taxonomic purposes, where the 16S rRNA gene sequence and the β subunit of RNA polymerase encoding gene *rpo*B are the molecular targets.

**Results::**

The genetic elements classified the subspecies to *B. licheniformis* 61.4%, in addition to, *Corynebacterium bovis* 29.8%. The somatic cell count (≤1×10^7^ cells/ml) and California mastitis test reactivity (+3 or +4) of milk clinically classified the she-camels population (n=100) under investigation into 50, 20, and 30 as healthy, subclinical, and clinical mastitic she-camels, respectively. During bacterial isolation, 80 species were noticed, of which 71.25% (57/80) and 28.75% (23/80) were Gram-positive and negative, respectively, in two clinical forms: Single (40%, n=16/40) and mixed (60%, n=34/40) bacterial infections. *In vitro*, 100% sensitivity for gentamycin (10 µg) and ofloxacin (5 µg) was noted; however, it was reduced to 50%. Moreover, during in vivo treatments, cloxacillin (5 µg) upraised as the most effective alternative with 90% sensitivity.

**Conclusion:**

Neither recurrent mastitis nor *Bacillus* species are thoroughly investigated with regard to reproduction performance in Egypt and the usefulness of these strains as antimastitis probiotics. Both persistent bacteremia and dormant endospores were formed but unaffected by standard schemes of antimicrobials injections which proposed the risk of pathogenic bacilli contaminating row milk from apparently healthy she-camel. The discrepancies between treatment results were induced by the resistance that started to develop by the organisms due to frequent and/or faulty use of applied antibiotics.

## Introduction

Mastitis has been estimated to affect more than 25% of lactating she-camel [1-7] with up to 70% milk loss [5-7], which is of significant on neonatal loss, and hence, birthing rates rarely exceed 40% in nomadic herds while 70% in more intensive camel herds [5]. There are limited data on the incidence of mastitis, endometritis, metritis, as well as the pathogenesis and treatment of uterine infections, probably due to the lack of routine laboratory investigations on Camelidae [5]. Although the official records of mastitis incidence, treatment cost, and antibiotic failure worldwide illustrated the inadequacy of the applied mastitis management protocols in lactating herds [1,2], FAO records considered those uterine infections to be the primary cause of mastitis and reproductive failure [6,8].

Camelids as a species have major economic importance in many areas of the world [1,2]. A common belief and practice among the Bedouin of the Sinai Peninsula are dependent on the healing properties of camel milk consumption toward any internal disease [3]. In the past, camel milk has been used to counter the effects of type 1 diabetes, milk allergies, Crohn’s disease, autism, dropsy, jaundice, chronic hepatitis, tuberculosis, asthma, anemia, lung- and spleen-related ailments, and piles. In addition, camel milk-based creams decrease inflammation of dermatological autoimmune diseases [3-6]. Recently, cases of mastitis refractory to any type of treatment have been reported frequently [9-11]. A wide range of Gram-positive spore-forming bacilli proved to be part of the teat normal microflora [12-15], commonly isolated from both raw and pasteurized milk as environmental commensals [14]. On the other hand, probiotic *Bacillus* species including *B. subtilis*, *B. cereus*, *B. alvei*, *B. megaterium, B. Clausii*, and *B. pumilus* are already marketed as gastrointestinal enhancers for both humans and animals and release antibacterial lipopeptides [11]. In addition, their bacteriocins are utilized as biocontrol agents of some infections as well as insect vectors [13]. Nonetheless, *B. cereus* was held responsible for hemorrhagic disease, when it was isolated from a 9-year-old she-camel that aborted; from the placenta and organs of the fetus (spleen, liver, and intestines) [15,16]. Since 1970s, *Bacillus licheniformis* proved to be involved in suppurative inflammation leading to abortion and/or mastitis, from mild to gangrenous, with frequent rapid fatalities [11]. Regardless that details of pathogenesis are yet unknown, previous outcomes were recorded in animal fed on poor quality silage and/or contaminated intramammary injections [15], where their pathogenicity is hooked on the discrepancy in host immunity [4,12]. Multifactorial mastitis has serious implications on public health [13], hence, serving as a media for transmission of various zoonotic diseases [13,14]. During infection, milk quality varies from normal to pink-tinged with blood in mild or early cases; later on, it became rusty-brown-colored discharge indicating the establishment of recurrent mastitis which is the typical prognosis of *B. licheniformis* infection [11,16].

The successful management of recurrent mastitis obligated intensive continuing doses of antibiotic during the treatment regimen, simultaneous with, periodical testing for bacteremia in milk and/or blood samples [4,8]. On the other hand, abuse use of antibiotics will enhance the development of drug resistance and failure to treat; at that point, the lactating animal will be culled from herd [7,15]. Public health and food safety associated with dairy products are reliant on milk quality (residuals and bacterial contents) and animal welfare, which necessities rapid and accurate identification of bacterial species [11]. Alternative analysis of species-specific stretches of the genomes, which are recommended for identification of atypical bacterial variants in phenotype, was developed [16]. Long ago, the 16S rDNA gene has been used for diagnostic purposes, developing phylogenetic relationships, in addition, for bacterial identification [12,16]. Several polymerase chain reaction (PCR)-mediated proof of identity/taxonomy were previously conducted using 16S rRNA gene sequence, the 16S-23S rRNA intergenic spacer region (ISR), and the β subunit of RNA polymerase encoding gene *rpo*B as molecular targets [8,16]. Despite that bioinformatics and genomes analytical reports had shown the low divergence (3-5%) between 16S rDNA genes sequences at subspecies level as the main disadvantage, of interest, high divergence at higher ranks in taxonomy is the main advantage in favors diagnostic purposes [8].

In Egypt, neither she-camels recurrent mastitis nor *Bacillus* species are thoroughly investigated. Considering the formerly mentioned issues, the present study was designed to isolate and characterize the pathogenic udder microbiota with regard to reproduction performance of she-camels in three governorates in Egypt: Giza, Marsa Matrouh, and Sharkia. Furthermore, to analyze the pathogenesis of she-camels recurrent mastitis due to antimicrobials resistance triggered by *Bacillus* spps.; pathogenic udder microbiota, which have potential public health risk, considering consumption of contaminated row milk from apparently healthy she-camel.

## Materials and Methods

### Ethical approval

All animal experimental procedures were in accordance with the ARRIVE guidelines and were carried out in accordance with the EU Directive 2010/63/EU for animal experiments and the National Institutes of Health guide for the care and use of laboratory animals (NIH Publications No. 8023, revised 1978). In addition, these adopted ethical guidelines are compiled with those of the national research center guidelines for the care and use of laboratory animals in Egypt.

### Animals population and geographical scope of the study

The present study was carried on she-camels (n=100) with a history of abortion and/or recurrent mastitis during 2 years, 2014 till 2016, collected from 10 localities in Marsa Matrouh, Giza, and Sharkia governorates in Egypt from smallholders farmers. Their age ranged 3-7 years in different lactation seasons. According to farmers’ interview and case records, animals were fed for high production and medical care was given to clinical abnormalities. The udder and teats of each female were examined by visual inspection and palpation for abnormal findings recording before sampling.

### California mastitis test (CMT)

In the present investigation, it was done before quarter-milk sampling. The results were read and evaluated with modifications according to Guliye *et al*. [17].

### Collection of milk samples

Aseptic samples were collected for bacteriological analysis using standard procedures [18]. The samples were transported to the laboratory at 4°C. Then, specimens were incubated for 2 h at 37°C for bacterial enrichment before undertaking the analysis [19].

### Milk somatic cell counts (SCCs)

Milk samples specified for SCC evaluation had been kept at 4°C and were analyzed within 24 h from the collection (SOMA-COUNT 150, Bentley, USA). The log10 (SCC) values had classified udders status into three categories: Normal, subclinical, and mastitic udders recorded SCC ≤4×10^5^, ≥4×10^5^, and ≥1×10^6^ cells/ml, respectively [17].

### Bacteriological survey on pathogens causing recurrent mastitis

Only the individuals reported with recurrent mastitis and/or abortion were included in the bacterio­logical survey, where milk samples were incubated both aerobically and under anaerobic conditions at 37°C [18,19].

### Antibiotic susceptibility patterns

Antibiotic susceptibilities of isolates were determined by disk diffusion method on Muller-Hinton agar (Oxoid) plates incubated under anaerobic conditions for 24-48 h [18,19]. Quinolones, beta-lactams, aminoglycosides, macrolides and sulfamides antibiotics have significant activity against both Gram-negative and Gram-positive microorganisms and thus were used in for during the treatment [18].

### Identification and characterization systems of *Bacillus* spp. isolates

#### Standard procedures for bacterial identification

The bacterial isolates were characterized based on phenotype, Gram-stain, and biochemical profiles [18,19]. For epidemiological records, each isolate was categorized as clinically relevant or a contaminant (pseudobacteremia) by clinical and laboratory criteria including; clinical signs, physical examination findings, body temperature, differential cell counts, SCC, histopathological findings of an aborted fetus, number of positive milk cultures, and response to treatment [17-20].

### Molecular identification system of bacterial genomics

#### Bacterial DNA isolation

Five to 10 colonies of each freshly streaked isolates were subsequently suspended in 180 µl Tris-EDTA buffer (Sigma Aldrich) containing 5 µl mutanolysin (10 U/µl, Sigma Aldrich). The extraction mixture [8] was added to each bacterial sample, incubated overnight at 56°C, and then DNA isolation was done by phase separation protocol [8,21-23]. The working DNA concentration was evaluated by NanoDrop 2000c (Thermo Scientific) and then adjusted to 100 ng/µl [24].

#### Internal quality control for molecular assays

Specific PCR assay was applied as a semi-qualitative control for the DNA extraction from camels’ milk/fetus samples. During this reaction a target fragment is amplified of about 400 bp from the 12S rRNA gene of the camel mitochondrial genome utilizing the L1091-F and H1478-R oligonucleotides [24,27] (Table-1) [3,8,24-27].

**Table-1:** List of oligonucleotides primers used in this study.

Target	Bacterial species	Primers	5`-sequences-3`	Fragment bp	References
16S rRNA	*Bacillus* and *Arcanobacteria*	16SBA-L	AGAGTTTGATCATGGCTCAG	1400	[8]
		16SBA-R	GTGTGACGGGCGGTGTGTAC		
	*Clostridia*	LPW58	AGGCCCGGGAACGTATTCAC	1200	[25]
		LPW81	TGGCGAACGGGTGAGTAA		
	*Listeria*	Lis-F	CAGCMGCCGCGGTAATWC	950	[3]
		Lis-R	CTCCATAAAGGTGACCCT		
	*Corynebacteria*	16SC-F	ACCGCACTTTAGTGTGTGTG	850	[26]
		16SC-R	TCTCTACGCCGATCTTGTAT		
16S-23S rRNA ISR	*Arcanobacteria*	16S-23S-ISR-F	GTTTTGCTTGTGATCGTGGTGGTTATGA	130	[8]
		16S-23S-ISR-R	AAGCAGGCCCACGCGCAGG		
*rop*B	*Bacillus* and *Corynebacteria*	C2700-F	CGTATGAACATCGGCCAGGT	450	[26]
		C3130-R	TCCATTTCGCCGAAGCGCTG		
12S rRNA	Internal control (mammals)	L1091-F	AAAAAGCTTCAAACTGGGATTAGATACCCCACTAT	380	[24,27]
		H1478-R	TGACTGCAGAGGGTGACGGGCGGTGTGT		

#### Identification of bacilli species by 16S rRNA genes identification system

PCR oligonucleotide primers sets were designed with reference and modifications of previous publications where the identification scope included all Gram-positive bacilli such as *Arcanobacteria*, *Corynebacteria*, *Listeria*, *Clostridia*, and *Bacillus* spp. [8,24-27]. PCR amplification and sequencing of the 16S rRNA genes were carried out by four sets of primers: 16SBA-L and 16SBA-R [8], LPW58 and LPW81 [25], Lis-F and Lis-R [2], and 16SC-F and 16SC-R [26] (Table-1). The four sets of primers were synthesized by Metabion International AG, Semmelweisstr, Germany.

#### Species-specific molecular virulence determinants

All Gram-positive bacilli isolates identified by 16S rRNA gene amplifications were further characterized by PCR-mediated species-specific virulence determinants. The 16S-23S rRNA ISR was *Arcanobacterium* species specific. *The* 16S-23S-ISR-F and **-**R is the primers pair that could amplify an expected size of 150 bp (Table-1). The *rop*B-C2700 and *rop*B-C3130 primers pair was specifically designed for *Corynebacteria* and *Bacillus* species identification through the sequence of 450 bp fragment of the β subunit of the RNA polymerase gene (*rop*B) (Table-1). All primers were synthesized by Metabion International AG, Semmelweisstr, Germany.

#### The cycling profiles of PCRs

All of the PCR assays were performed in 25 μl total volume mixtures including 3 μl of DNA (100 ng/µl), 50 pM/µl of each primer, 10 mM dNTP, 25 mM MgCl_2,_ 5 U/reaction Taq DNA polymerase, and nuclease-free water to complete the volume of each reaction (Qiagen). PCRs were performed in a PTC-100™ Thermal Cycler (MJ Research Inc., USA). A reagent blank was used as control negative. Pure colonies of *Listeria monocytogenes*, *Corynebacterium pyogenes*, *Arcanobacterium pyogenes, B. subtilis* and *Clostridium perfringens* were utilized as controls positive [24,27]. The PCR programs for each primers pairs were carried out as described elsewhere [3,8,24-27].

Amplified products were electrophorized in 1.5% agarose gels in TBE buffer (Sigma Aldrich), stained with ethidium bromide (Sigma Aldrich) [22], and then analyzed using Image Lab (version 5.2, Bio-Rad). Gene Ruler 100 bp Plus DNA ladder, ready to use (Thermo scientific, Fermentas), was used with each gel.

#### Sequencing of PCRs products

Each amplicons type was purified for sequencing using the QIAquick Spin PCR Purification kit (Qiagen) according to the manufacturer’s instructions. Sequencing reactions were performed with the dye-terminator DNA sequencing kit on an ABI 3100 DNA sequencer (Applied Biosystems, USA), as described by the manufacturer. Each sequencing reaction was repeated at least 3 times in both directions before being accepted for analysis by multiple sequence alignment using the Clustal^®^W program [28].

## Results

### Udders health impression and SCC analysis

The visual inspection of the 100 she-camels udders did not display systemic symptoms; in addition, the milk samples were obviously normal. After analysis of both CMT and SCC scores, sampled population was divided into 50, 20, and 30 females as healthy, subclinical mastitic, and clinical mastitic she-camels, respectively. Samples of recurrent mastitic females possessed wide SCC range (≤1×10^7^ cells/ml), while CMT reactivity was +3 or +4. Abortion was previously reported for 25 individual according to records of the examined localities. For SCC controls, ten normal bacteriologically negative milk samples of healthy she-camels were randomly selected (SCC ≤4×10^5^ cells/ml).

### Prevalence of clinical infections in recurrent mastitic she-camels

Forty out of the examined udders, the subclinical/clinical mastitic females=50, revealed 80 bacterial isolates on stroked agar plates; the remaining 20% represent samples with no bacterial growth despite the indications of mastitis. On Gram-staining, Gram-positive and Gram-negative bacterial isolates represented 71.25% (57/80) and 28.75% (23/80), respectively, were counted. Contagious, environmental, and commensal bacterial pathogens were detected from recurrent mastitic she-camels in two clinical forms: Single (40%, n=16/40) and mixed (60%, n=34/40) bacterial infections.

### Phenotypic characteristics of anaerobic bacterial isolates

Within Gram-positive isolates which represented 71.25% (57/80), colonies with the typical mucoid appearance characteristic of pyogenic bacterial species were further characterized. The anaerobic Gram-positive bacilli isolated, whose characterized by pinpointed, grayish, glistening, circular, convex, smooth, opalescent colonies and surrounded with a narrow sharp hemolytic zone (β-hemolytic colonies), On Gram-staining, these colonies were small curved rod-shaped (bacilli), in addition, oval subterminal spores under the ×40 microscopic examination were identified.. The phenotypically classified bacterial species were investigated on the genetic structural level by 16S rRNA identification system. The *Bacillus* and *Corynebacterium* spp. have 61.4% (35/57) and 29.8% (17/57) detection percentage, respectively, within obtained colonies.

### Antibiogram patterns

**Figure-1:**
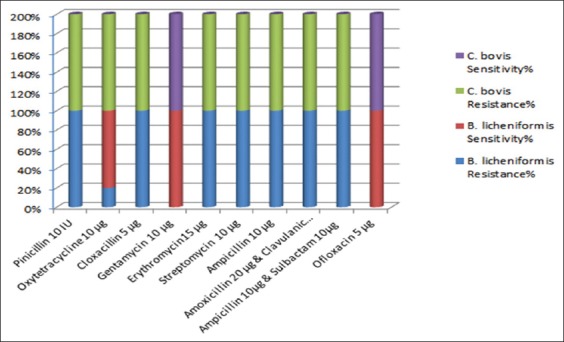
The *in vitro* antibiotic reactivity’s of the two predominant Gram-positive bacilli-purified colonies of *Bacillus* *licheniformis* and *Corynebacterium bovis* from lactating she-camels milk samples.

**Figure-2:**
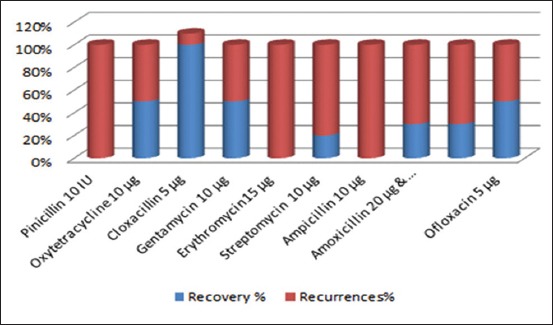
The recovery profile of the lactating she-camels in response to treatment with antibiotics against predominant bacterial isolates from milk microbiota of subclinical and/or clinical cases.

The *in vitro* antibiotic reactivity of the two main Gram-positive bacilli purified colonies of *B. licheniformis* and *Corynebacterium bovis* from she-camels milk samples is illustrated in Figure-1. On the other hand, the recovery profile of the she-camels with subclinical and clinical mastitis in response to treatment (*in vivo*) with those *in vitro* evaluated antibiotics is illustrated in Figure-2.

### 16S rRNA genes sequence-based molecular identification of *B. licheniformis* and *C. bovis*

**Figure-3:**
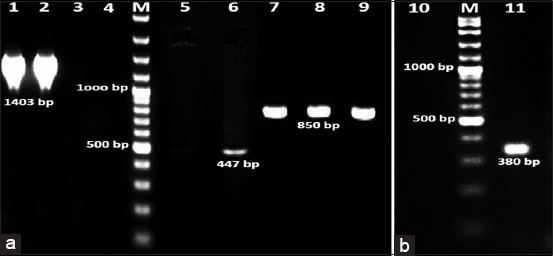
Molecular identified *Bacillus* *licheniformis* and *Corynebacterium bovis* pure colonies isolated from recurrent mastitic she-camels udders represented by polymerase chain reaction (PCR) products; (a and b) lane 1 and 2: 1403 bp bands, and lanes 8 and 9: 850 bp bands are amplifications of 16S rRNA genes. Lanes 3, 4, 5, and 10: Are blank controls negative. Lanes 2 and 7: Are PCR products of controls positive *Corynebacterium pyogenes* and *Bacillus subtilis* strains. Lanes M: are Gene Ruler 100 bp Plus DNA ladder.

Positive results with bands possessed the molecular sizes 1403 bp and 850 bp for 16S rRNA genes specific for *Bacillus* and *Corynebacteria* spp., respectively, were visible in designated isolates lanes of DNA agars stained with ethidium bromide (Figure-3a). Moreover, 447 bp fragments were amplified that are *rop*B genes’ specific for *Bacillus* and *Corynebacteria* spp. (Figure-3a). Negative results were obtained from blanks (Figure-3a), in addition, expected amplification was obtained with the reference strain in each PCR trials as well as from internal control, 380 bp bands (Figure-3b).

Sequences of the amplified 16S rRNA and *rop*B genes were compared with those available in GenBank using gapped BLASTN software [29]. BLASTN analysis of the aligned sequences of the isolates showed 99-96% identity with the 16S rRNA and *rop*B genes sequence of *B. licheniformis* (accession no. AF172323). Phylogenetic analyses grouped present isolates into branches with *B. licheniformis*, strains ATCC 14580 (CP000002), WX-02 (CP012110), BL1202 (CP017247), and HRBL15TD17 (CP014781) in one clade separated from other species. According to the incidence of infectious bacterial isolates, the research team considered *C. bovis* secondary to *B. licheniformis* in mastitis and/or abortion induction; their results were neither illustrated nor discussed.

### Biochemical profile of purified colonies of *B. licheniformis* field isolates

The biochemical profile tests were utilized to confirm the results of identified subspecies as *B. licheniformis* and *C. bovis*. The *B. licheniformis* colonies had positive results in glucose, cellobiose, galactose, salicin, xylose, raffinose, citrate, nitrate, and casein hydrolysis tests. On the other hand, *B. licheniformis* colonies gave negative results in mannose, urease, indole, VP, and oxidase tests (*C. bovis* results are not shown).

## Discussion

According to previous publications, it is the first detailed study of *B. licheniformis* infection in the she-camels with recurrent mastitis and/or abortion with sepsis in Egypt [8,30]. Several PCR-mediated species-specific proofs of identity were applied during this investigation for diagnostic and taxonomic purposes. The 16S rRNA gene sequence and the β subunit of RNA polymerase encoding gene (*rpo*B) were the targets during the present study (Figure-3). These genetic elements had classified the Gram-positive bacilli isolated species/subspecies to *B. licheniformis* 61.4% (35/57) and *C. bovis* 29.8% (17/57); the alignment sequence similarities recorded >97% [29]. The alignment clades supported the traditional phenotypic, biochemical, and Gram-stain identification of these isolates; yet, it indicated mixed populations per she-camel herd and/or locality. According to the incidence of infections, *C. bovis* is secondary to *B. licheniformis* in mastitis and/or abortion induction; therefore, their results were neither illustrated nor discussed. The *C. bovis* low incidence of infection (29.8%) resembled the findings reported by Abdelgadir [14] and Alqurashi *et al*. [15] and yet opposed those of Suheir (2004) **[**31]. Meanwhile, Ramadan *et al*. [29] and Hafez *et al*. [32] declared that the main cause of all types of she-camel mastitis is *Bacillus* spp., precisely *B. cereus*. The percentage of recurrent mastitic (subclinical/clinical) she-camels infected with *Bacillus* spp. was found higher than the records of Hoa *et al*. [33] who reported lower incidence (39%). The present investigation had confirmed the virulence of *B. licheniformis* (61.4%) that flourished in the she-camel udder, then ascend to the uterus causing abortion with sepsis, which is a series prognosis of the consequences to be more pathogenic than those previously report by others [2,6,15,16].

Bacillus species, which are not identified as part of the normal gastrointestinal and/or teat flora, have been used as gastrointestinal probiotics, and their effectiveness seems to be achieved in the course of passage without germination [34]. Genus *Bacillus* had inhibitory lipopeptides that are effective to a wide variety of clinical pathogens including the common mastitis-associated species, even strains of methicillin-resistant *Staphylococcus aureus* and vancomycin-resistant enterococci [18]. Difficulties associated with the production of these inhibitors have been encountered by many research groups [34]. The antimicrobials release is dependent on biofilm-specific signaling; the most common and preferred growth mode associated with critical cell densities in cultures and direct air exposure to trigger the expression of corresponding operon [35]. Similarly, *B. licheniformis* was found to produce antimicrobials when being growing as a biofilm in an air-membrane surface bioreactor [35,36]. In the present study, the isolated *B. licheniformis* appears to be part of the udder flora, a finding which is in agreement with the previous reports [4] and documented by being commonly isolated from raw and even pasteurized milk [36-37]. The proposal that this field isolate could fulfill udder probiotics potential, will depend on the outcome of future-controlled field trials simultaneous with genome complete annotation.

The *in vitro* antibiotic sensitivity experiments indicated that both *B. licheniformis* and *C. bovis* isolates were highly sensitive to gentamycin and ofloxacin (100%), which are partially in agreement with Dewani [36] but in distinction to the findings of Fazlani *et al*. [7] and Rind and Khan [37] who havetested previously the same drugs on the same microbial species (Figure-1). On the other hand, the *C. bovis* showed high resistance to oxytetracycline in contrast to *B. licheniformis* which was redeploy sensitive (80%) (Figure-1). All these results were dissimilar to those reported previously by Fazlani *et al*. [7]. Conversely, Tumini *et al*. [11] reported *B. licheniformis* spores’ highest susceptibility inducing complete recovery with quinolones antibiotics (65 mg/L of ciprofloxacin, 63 mg/L of danofloxacin, 109 mg/L of enrofloxacin, 101 mg/L of marbofloxacin, and 109 mg/L of sarafloxacin) that were in match with maximum residue limits worldwide [18,38,39]. In addition, adequate cross-sensitivity was noted from classified bacterial species to beta-lactams group (amoxicillin, ampicillin, cloxacillin, oxacillin, penicillin “G”, cefoperazone, ceftiofur, and cephalexin), aminoglycosides group (kanamycin, neomycin, and streptomycin), macrolides group (erythromycin, tylosin, and tilmicosin), and sulfamides group (sulfadiazine, sulfadimethoxine, sulfamethoxazole, and sulfathiazole). Nagel *et al*. [39], on the other hand, noted higher sensitivity yet with recurrences of infection with penicillin G, oxytetracycline, erythromycin, spiramycin, tylosin, and sulfamides. In the present study, the *in* *vivo* observations proved that in complete recovery of mastitis (50%) with recurrences incidences were noted with gentamycin and ofloxacin administration (Figures-1 and 2). In continuant, oxytetracycline induced similar recovery percentage (50%), in contrast to the *in vitro* observations (Figures-1 and 2). Nonetheless, cloxacillin intramammary injections revealed nearly complete recovery (90%) (Figure-2). The *in* *vitro* results are organism-based genetic constituent; nonetheless, the *in* *vivo* responses are the sum of the udder immune reactions in collaboration with the normal flora antibacterial lipopeptides and bacteriocins releases [11]. Undoubtedly, the investigated she-camels persistently harbored a heterogeneous population of inhibitory *B. licheniformis* within their udder microflora; moreover, the predominant inhibitory strains frequently differed from one sampling session to the next and even within a single herd [11,18]. In addition, results indicated that the mode of action of released microbial inhibitors of *B. licheniformis* field isolates antipathetic gentamycin and ofloxacin, yet synergism with cloxacillin active principles that were intramammary injected [38-40]; hence, the discrepancy in recovery results between she-camels clinically treated.

Epidemiologically, the case reported here is of particular interest for the following reasons: (a) The complains started with subclinical mastitis, later on, the clinical picture developed to recurrent mastitis and/or abortion with sepsis, (b) most cases of virulent *B. licheniformis* reported in the literature were observed in immunocompromised human patients but not isolated from camels lactating herds, (c) recurrent isolation of the typical isolate per she-camel, which was genes’ sequences’ similarity % dependent, indicated that the designed *B. licheniformis* is capable of persisting as numerically significant member of the teats and/or uterine microflora for extended periods, (d) virulent *B. licheniformis* isolates, were not detected in blood between the mastitis recurrences, they are likely to be persistent in other tissues; in the bone marrow and/or supramammary lymph node, (e) they remained dormant within the predeliction tissues, thus periodically germinating spores were able to cause recurrent systematic infections in lactating she-camels; hence, considering the immunosuppressive status induced by the stress of milk production and ecological inferior conditions, and (f) the abuse injections of antimicrobials subsequentily, their residuals in milk and milk products are biohazards to public health where raw milk consumption is still practised in part of the Egyptian society.

## Conclusion

PCR-mediated amplification then sequencing of 16S rRNA and *rpo*B genes proved their successful to classify the she-camels Gram-positive bacilli to *B. licheniformis* which confirmed their diagnostic and taxonomic features. To the best of our knowledge, the present study is the first detailed phenotypic and genotypic characterization of *B. licheniformis* isolates from recurrent mastitis and/or abortion with sepsis. However, at present, nothing is known about the route of infection or the zoonotic potential, these strains might have for the camels’ owners and/or raw milk consumers. Transient colonization with bacillus probiotics has been reported to produce a beneficial outcome in humans and animals immunity to infections. The potential for being used as antimastitis probiotics seems prospective and indeed easier, hence, comprises a considerable proportion of the normal flora of the healthy teat and that the activity of some of these strains is very broad against Gram-positive mastitis pathogens.

## Authors’ Contributions

EKM offered the control positive strains applied during laboratory study. NATA and DS conceived the study, carried out the laboratory work, and analyzed the data. NATA performed the fieldwork, collected the samples, and drafted the manuscript. All authors read and approved the final manuscript.
